# Physiotherapy-assisted overground exoskeleton use: mixed methods feasibility study protocol quantifying the user experience, as well as functional, neural, and muscular outcomes in children with mobility impairments

**DOI:** 10.3389/fnins.2024.1398459

**Published:** 2024-07-31

**Authors:** Stefanie S. Bradley, Ledycnarf Januario de Holanda, Tom Chau, F. Virginia Wright

**Affiliations:** ^1^Bloorview Research Institute, Holland Bloorview Kids Rehabilitation Hospital, Toronto, ON, Canada; ^2^Institute of Biomedical Engineering, University of Toronto, Toronto, ON, Canada; ^3^Department of Physical Therapy, University of Toronto, Toronto, ON, Canada

**Keywords:** lower limb exoskeleton, overground gait training, cerebral palsy, GMFCS IV, robotic training, pediatric, mobility, physiotherapy

## Abstract

**Background:**

Early phase research suggests that physiotherapy paired with use of robotic walking aids provides a novel opportunity for children with severe mobility challenges to experience active walking. The Trexo Plus is a pediatric lower limb exoskeleton mounted on a wheeled walker frame, and is adjustable to fit a child’s positional and gait requirements. It guides and powers the child’s leg movements in a way that is individualized to their movement potential and upright support needs, and can provide progressive challenges for walking within a physiotherapy-based motor learning treatment paradigm.

**Methods:**

This protocol outlines a single group mixed-methods study that assesses the feasibility of physiotherapy-assisted overground Trexo use in school and outpatient settings during a 6-week physiotherapy block. Children ages 3–6 years (*n* = 10; cerebral palsy or related disorder, Gross Motor Function Classification System level IV) will be recruited by circle of care invitations to participate. Study indicators/outcomes will focus on evaluation of: (i) clinical feasibility, safety, and acceptability of intervention; (ii) pre-post intervention motor/functional outcomes; (iii) pre-post intervention brain structure characterization and resting state brain connectivity; (iv) muscle activity characterization during Trexo-assisted gait and natural assisted gait; (v) heart rate during Trexo-assisted gait and natural assisted gait; and (vi) user experience and perceptions of physiotherapists, children, and parents.

**Discussion:**

This will be the first study to investigate feasibility indicators, outcomes, and experiences of Trexo-based physiotherapy in a school and outpatient context with children who have mobility challenges. It will explore the possibility of experience-dependent neuroplasticity in the context of gait rehabilitation, as well as associated functional and muscular outcomes. Finally, the study will address important questions about clinical utility and future adoption of the device from the physiotherapists’ perspective, comfort and engagement from the children’s perspective, and the impressions of parents about the value of introducing this technology as an early intervention.

**Clinical trial registration:**

https://clinicaltrials.gov, identifier NCT05463211

## 1 Introduction

While wheelchairs are essential for supporting mobility and participation in the daily activities of individuals who have severe motor impairments (Gibson et al., 2012), they do not offer supported upright positioning of the body. Many individuals with limited mobility use a combination of assistive devices for different intentions or circumstances, such as wheelchairs, standing frames, and supported walkers (Moen and Østensjø, 2023). Walking (or assisted standing) confers a multitude of health benefits including improved bone health (Kim et al., 2017), respiration (Lee and Kim, 2014), circulation (Eng et al., 2001), urination (Houle et al., 1998; Walter et al., 1999), bowel function (Walter et al., 1999), joint range of motion (Paleg et al., 2013), sleep (Eng et al., 2001), as well as psychosocial (McKeever et al., 2013; Livingstone and Paleg, 2023) and mental health benefits (Kenyon et al., 2021). Growing recognition across rehabilitation sectors about the possibility of technology to facilitate enhanced upright mobility and independence has stimulated the engineering advancement of assistive gait devices from simple wheeled walkers (Tao et al., 2020), to supported stepping devices (Livingstone and Paleg, 2023), to non-robotic mechanically facilitated walkers (Wright and Jutai, 2006; Paleg and Livingstone, 2015), to treadmill mounted (tethered) gait trainers (Cherni and Ziane, 2022), and most recently to powered exoskeletons that move overground (Owens et al., 2020).

Overground lower-limb powered exoskeletons afford earth vertical weight-bearing positioning that is coupled with augmented mobility (De Luca et al., 2019), giving novel upright movement opportunities when foundational gross motor skills (e.g., learning to walk) are delayed or when disease or injury has resulted in loss of independent walking abilities (Hunt, 2021). These exoskeletons can be used in conjunction with other gait aids such as wheeled walkers, or tethered on a treadmill (Kim et al., 2021) to fit the individual’s support needs. They can also be built as freestanding robotically guided mobile walking frames (De Luca et al., 2019; Sarajchi et al., 2021). The added value of exoskeleton use within gait-based physiotherapy sessions is that they provide: the option of hands-free body weight support (thereby freeing the arms/hands for other activities), sensors that respond to biological feedback, natural joint movements and activation of weak or spastic muscles by modulating forces on body segments, normalization of the gait cycle by standardizing step length and range of motion, reduced cost of walking to allow meaningful periods of exercise during intervention sessions, quantified session progress to provide real-time feedback, and supported use of motor learning protocols (intensity, repetition, variability, and task-specificity) to optimize gait training (Molteni et al., 2018; De Luca et al., 2019).

Increased clinical adoption of mobile exoskeletons such as the Angel-legs (Angel Robotics Co., Ltd., Seoul, Korea), ReWalk (ReWalk Robotics Inc., Marlborough, MA, USA), and Ekso (Ekso Bionics, Richmond, CA, USA) has occurred over the last decade in adult rehabilitation, and there is an emerging body of evidence of the physical and health benefits of exoskeleton use by adults with neuromotor conditions (Louie and Eng, 2016; Bruni et al., 2018; Rojek et al., 2020; Karunakaran et al., 2021; Rodríguez-Fernández et al., 2021). However, development and access to smaller sized exoskeletons for pediatric populations, and associated clinical experience with using them, currently lags far behind that of adults (Fosch-Villaronga et al., 2020). As a result, there is limited knowledge on the neurological and neuromuscular effects of exoskeleton use in children, as well as the training considerations and user perspectives that are essential to facilitate best practice use. The majority of research to date on robotic walkers in pediatrics has been with treadmill-based tethered models such as the Lokomat robotic gait trainer (Hocoma AG, Volketswil, Switzerland) (Aurich-Schuler et al., 2015; Cumplido et al., 2021; Kim et al., 2021; Cherni and Ziane, 2022). While tethered exoskeletons offer weightbearing and gait training benefits (Kim et al., 2021), they do not provide the functional and participation opportunities obtained from the added experience of moving overground with mobile exoskeletons like Ekso, Trexo pediatric frame-mounted exoskeleton (Trexo Robotics, 2022), or similar devices.

The Trexo Plus (hereafter referred to as the Trexo), designed specifically for use with children with motor impairments, has been commercially available since 2017. Thus far it has been investigated in the context of home use with children’s caregivers operating the device after orientation provided by the Trexo team (Diot et al., 2021, 2023). Benefits in the areas of sleep quality, bowel function, postural function, and positive affect associated with Trexo use have been documented (Diot et al., 2023). At the time of writing, Trexo use is being evaluated in a follow-up crossover feasibility RCT (4-week Trexo home program [prescribed/taught by a PT and facilitated by the child’s caregiver 4–5 times/week] compared with a 4-week identical frequency functional therapy program [prescribed/taught by a PT and facilitated by the child’s caregiver]) (McCormick et al., 2023).

There are no published studies to date that focus on integration of the Trexo into clinical settings, specifically physiotherapist-led therapy sessions. There is evidence though of adoption-related challenges for rehabilitation teams using exoskeletons in adult rehabilitation, such as extensive knowledge requirements and hands-on skill demands to handle the operation of these advanced new technologies for competent goal-based therapeutic use, while ensuring patient safety and comfort (Read et al., 2020). There is also the need for strong teamwork within a facility to collaboratively develop therapy protocols for each patient that will optimize outcomes (Gilardi et al., 2020).

Paradigmatic shifts in thinking about possible walking-based outcomes for minimally ambulatory children (van Hedel et al., 2016; Livingstone and Paleg, 2023) are occurring in tandem with assistive device developments. For example, typical physiotherapy and occupational therapy goals for children with cerebral palsy (CP) who are minimally ambulatory or non-ambulatory (i.e., Gross Motor Function Classification System [GMFCS] levels IV and V respectively) have traditionally focused on achieving their highest degree of independence within the context of their physical constraints (Goswami et al., 2021). The recent introduction of overground robotic devices (such as the Trexo) has been encouraging a transition in practice toward providing more active focus on upright assisted walking within home and community environments, especially in children’s younger years, using a physiotherapy intervention approach that is based on principles of motor learning and neuroplasticity. However, from a best practice perspective, there is an urgent responsibility to gather clinical evidence both on intervention processes and associated outcomes before making them a part of regular clinical care (Phelan et al., 2015).

The primary aim of this study is to investigate the feasibility, user perspectives, and body-wide outcomes associated with institutionally based overground exoskeleton gait training in children 3–6 years of age with a functional presentation of GMFCS level IV. The Trexo will be the overground lower limb exoskeleton used. It is listed as a Class I medical device by Health Canada and Class II medical device by the FDA.

The study aims will be completed through a study protocol with the following objectives:

i) Assess aspects of clinical feasibility, safety, and acceptability of Trexo gait training within an outpatient center and school
ii) Capture the Trexo user experience of children and physiotherapists during exoskeleton gait training, as well as physiotherapists’ and parents’ perspectives of outcomes associated with use
iii) Assess motor and functional outcomes pre/post Trexo gait training (including any carryover effects)
iv) Examine brain anatomy and brain connectivity pre/post Trexo gait training
v) Evaluate muscle activations, particularly indicating muscle fatigue, during Trexo gait training (compare with muscle activations during use of regular assistive mobility devices)
vi) Capture heart rate and heart rate variability during Trexo gait training (compare with heart rate during use of regular assistive mobility devices).

## 2 Methods and analysis

### 2.1 Study design

**FIGURE 1:**
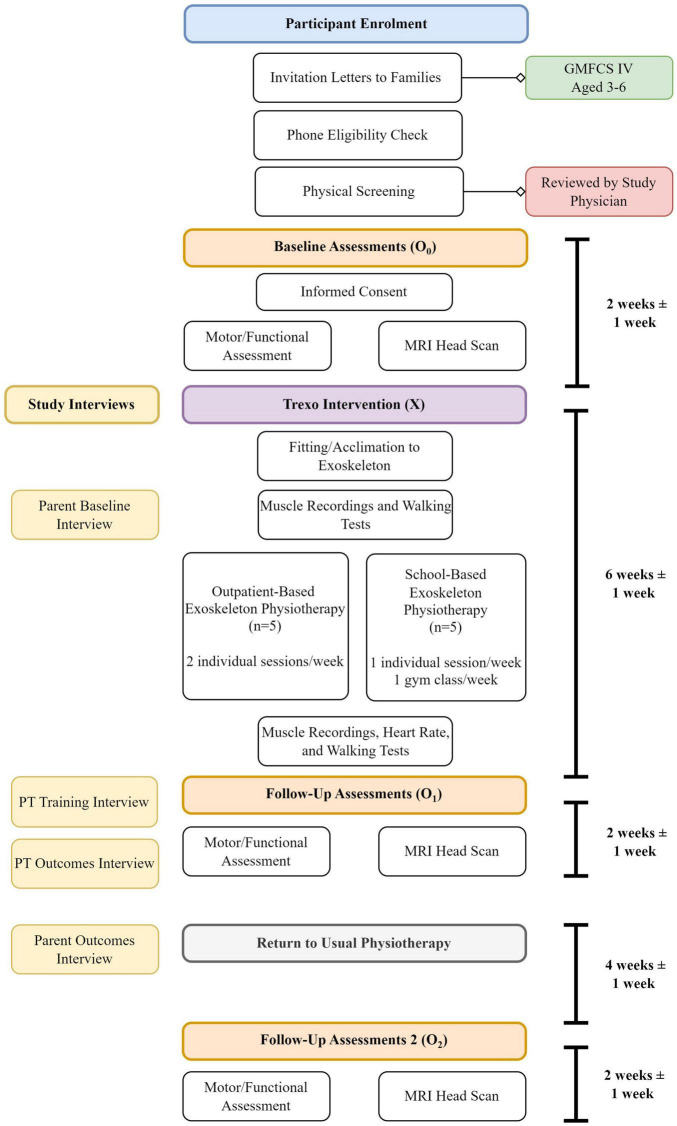
Flow chart of study visits and timeline, reflecting O_0_ X O_1_ O_2_ design.

This mixed methods feasibility study protocol [phase IIa; Orbit Model (Czajkowski et al., 2015) uses an **O_0_ X O_1_ O_2_** design (Figure 1), with participants (*n* = 10) acting as their own controls (within a longitudinal intervention consisting of O_0_, O_1_, and O_2_ = study assessment phases, and X = physiotherapy intervention with the Trexo]. The protocol follows Standard Protocol Items: Recommendations for Interventional Trials (SPIRIT Checklist; Chan et al., 2013a,b). All study team members’ roles are outlined in Table 1.

**TABLE 1:** Research team member designations and corresponding roles in the study.

Designation	Role
Principal investigators	Responsible for study oversight and adverse event management.
Study researchers	Non-PT team members that are responsible for coordinating recruitment, scheduling, non-PT data collection, and data analysis.
Study physician	Center-affiliated pediatrician that helps with diagnostic expertise, recruitment, eligibility, and adverse event management.
Treating PTs/PTAs	Physiotherapy team members that are Trexo-trained and complete the 12 Trexo (twice weekly) intervention sessions.
PT assessors	Independent PTs (different from treating PTs) that complete the physical screening and motor/functional assessments.
Study interviewer	Independent team member that hosts all Zoom-based interviews.
MRI technician	Centre-affiliated technician that operates MRI scanner.

During ‘**O’ phases (pre/post intervention)**, participants will do their usual physiotherapy regimen (with their usual assistive device, where applicable) and associated home program, and will undergo the set of study assessments (gross motor/functional assessments and neuroimaging) in this approximately 2–3-week period pre- and post-Trexo use (Phase X). One-month post O_1_, participants will have one final motor/functional assessment and neuroimaging visit (O_2_). They will continue with their usual physiotherapy regimen during the month between O_1_ and O_2_.

**Intervention ‘X’ phase** will consist of the Trexo physiotherapy block: physiotherapy sessions twice weekly (30-min sessions excluding initial set-up time) for 6 weeks provided by a study-trained PT/Physiotherapist Assistant (PTA) team within a goal-based training program that is grounded in motor learning principles. Trexo physiotherapy will be integrated within a school-based program for half of the study cohort (*n* = 5), and within an outpatient program for the other half (*n* = 5). Each child will receive 1–2 acclimatization/fitting sessions in the Trexo before starting physiotherapy sessions. Walking tests and muscle recordings at the start and end of this phase will be done in the Trexo and in the child’s usual non-robotic wheeled walker (if they use one) for comparison and for assessment of potential carryover effects from Trexo use to their wheeled walker.

Independent PT assessors (different from the intervention PTs) will perform motor/functional assessments, with the same assessor assigned to a child for each assessment. They will be blinded to the results of their previous assessment(s). Treating Trexo PTs and PTAs may choose to share their experience of using the Trexo as an intervention via optional qualitative interviews pertaining to: (i) their Trexo training/learning process, and (ii) the treatment process specific to each of the children who is assigned to them. Parents may choose to share their expectations and impressions of the Trexo treatment block via optional qualitative interviews pertaining to: (i) baseline study expectations, and (ii) post-intervention impressions and any associated outcomes of their child’s use.

### 2.2 Participant eligibility

#### 2.2.1 Age range justification

We decided to acquire a medium-sized Trexo, which fits children aged 3–6 years old (height and weight requirements). We aim to work with children at an early intervention point where there is still considerable developmental potential for change and capitalize on the developing brain’s neuroplastic nature (Novak et al., 2017). According to GMFM Motor Growth Curves for children with CP (GMFCS IV) there is still potential for motor gains for children under the age of 6 (Rosenbaum et al., 2002).

#### 2.2.2 Inclusion criteria

(a) Age 3–6 years inclusive at the time of receiving the study invitation; (b) mobility impairment caused by a non-progressive neuromuscular disorder, classified as **GMFCS Level IV** or equivalent: uses a wheelchair (pushed by others or powered) most of the time, and walking is very limited even with use of assistive devices (Palisano et al., 2008) and able to sit on chair but need adaptive seating for trunk control and to maximize hand function, can move in and out of chair with assistance from an adult or a stable surface to push or pull up on with their arms, can walk no more than short distances with a maximum support walker/stepping device and caregiver assistance (Rosenbaum et al., 2008); (c) weight: 20–100 lbs to fit within our medium size Trexo; (d) leg length with specific measurement of the hip to the knee of 17–27 cm and knee to floor of 18–32 cm while wearing shoes; (e) able to indicate pain, fear, or discomfort verbally or non-verbally; (f) able to respond to one or two-step commands; and (g) at least 2 months after any lower limb Botulinum Toxin injections. Children may have a maximum support manual walker (e.g., a supported stepping device such as the Rifton Pacer Gait Trainer^1^ - a wheeled “walking” frame or support walker that provides trunk and pelvic support and has a soft strap or solid seat and arm support as needed) that they use as their gait device at home/school, but this will not be necessary to be eligible to participate in the study.

#### 2.2.3 Exclusion criteria

(a) As per the Trexo Plus Operations manual (Trexo Robotics, 2022), unless cleared by study physician: knee flexion contracture > 20°; knee valgus > 40°; hip extension < −10°; hip subluxation > 40%; (b) dynamic spasticity or behavioral concerns that interfere with the use of the Trexo; (c) weight-bearing restrictions (d) osteogenesis imperfecta; (e) orthopedic surgery within the last 6 months (if muscle) or 12 months (if bone), or planned within the next 6 months; (f) seizure disorder that’s not controlled by medication; (g) unable to pass MRI screening; (h) involved in another interventional study (reviewed on a case-by-case basis); (i) received robotic exoskeleton training in the past; (j) neurological, respiratory, cardiac, and orthopedic medical conditions that would restrict physical activity as reported by parents; (k) open skin lesions or vascular disorders of the lower extremities; and (l) not able to discontinue Botulinum Toxin injections for 6-week period during study intervention.

#### 2.2.4 Sample size justification

The planned sample size of 10 children for this feasibility study will be sufficient to give an initial group picture through descriptive statistics and summary graphs of feasibility indicators and quantitative outcomes. This aligns with other quantitative pediatric therapy technology-based intervention studies that have successfully provided meaningful feasibility study results with 4 to 20 participants: (Richards et al., 1997; Wallen et al., 2008; Weightman et al., 2011; Radtka et al., 2013). This sample size will also elucidate how a single robotic assistive device may be shared (i.e., fitting adaptations to support use) among multiple children for use as a physiotherapy intervention within a center.

For the qualitative user perspective data, there is no agreed-upon sample size to achieve saturation in qualitative research (Saunders et al., 2018), instead depending collectively on sample homogeneity, interaction quality, and theoretical framework (Malterud et al., 2016).

Previous qualitative descriptive studies with children, parents, or clinicians reporting on user experiences of applying new technology or outcome measures have produced meaningful results with samples of 5 to 13 participants (Rich et al., 2014; Beveridge et al., 2015; Phelan et al., 2015; Kahlon et al., 2019; Vaughan-Graham et al., 2020; Giancolo et al., 2022; Hadj-Moussa et al., 2022; Torchia et al., 2023).

### 2.3 Participant enrolment

#### 2.3.1 Invitation letters from circle of care

Pediatricians, PTs, and clinic staff affiliated with the hospital-based outpatient program and the school integrated education therapy program will share study invitation letters with parents of the children on their caseload who meet the main eligibility criteria. Interested parents will have an initial phone conversation with the study’s research coordinator who will provide them with full details on the study, and if they are interested in moving ahead, will then review the study’s basic eligibility questions. If these criteria are met, they will be added to a candidate list that will be capped by a predetermined date deadline. If the list exceeds capacity, names will be drawn by a random number system (randomizer.org) to schedule an in-person physical screening visit to confirm eligibility.

#### 2.3.2 Physical screening

A PT assessor will perform the child’s in-person physical screening following receipt of written informed consent from the parent, and will document the following: height (body and leg lengths), range of motion, orthopedic/medical suitability, cognitive ability to participate in physiotherapy, current home program, method of communicating discomfort or pain, and parent’s willingness to commit to the treatment frequency and to refrain from commencing other new therapies during O_0_, X, and O_1_ phases the study. The study physician will review the results of this screening and confirm the child’s eligibility to proceed with the study.

#### 2.3.3 Informed consent

Informed consent will be obtained from: child’s parent, child (assent; contingent on capacity assessment determination prior to the consent session) for the Trexo intervention and associated assessments, PT/PTAs for study interviews (post child’s Trexo use), and parent for study interviews (pre and post child’s Trexo use). Consent for neuroimaging will be obtained separately.

### 2.4 Study intervention

#### 2.4.1 Trexo plus pediatric exoskeleton

**FIGURE 2:**
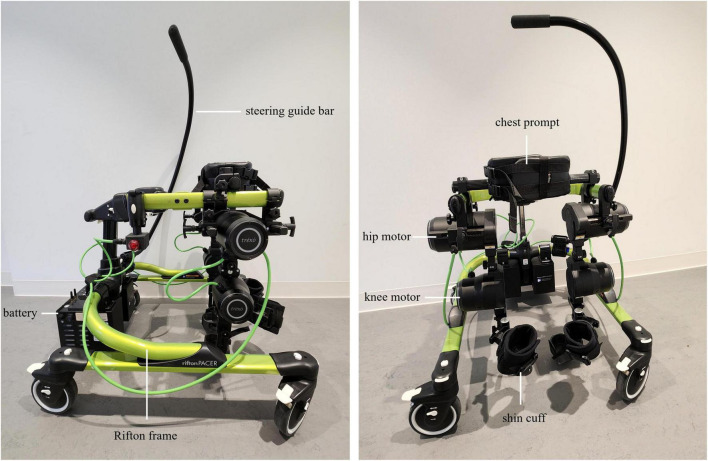
Trexo Plus pediatric lower-limb exoskeleton, mounted on a medium-sized Rifton Pacer Gait trainer. Lateral **(left)** and anterior **(right)** views are shown. Exoskeleton battery, motors at the hip and knee, and shin cuffs are indicated. Rifton frame, steering guide bar, and chest prompt are also indicated. Trexo orientation is set-up for child to be facing out of the open side of Rifton frame. Tablet interface for controlling the robotic legs is not shown.

The Trexo Plus lower limb exoskeleton (Figure 2) comprises powered orthotic legs which work cohesively within a Rifton Pacer wheeled walker that has been adapted to accommodate the exoskeleton attached to its frame. The Trexo’s robotic legs are a multi-joint system with two actuated degrees of freedom per lower limb for hip flexion-extension and knee flexion-extension. This design makes the device responsive to the child’s ability to initiate steps, and able to provide proportional support needed for leg movement as provided through more mechanically active “endurance mode” or more passive “strength mode”. It can also be used in “standing mode” for upright activity facilitation. Trexo settings are adjusted via a user-friendly tablet interface that monitors walking time, step count, and initiation (Trexo Robotics, 2022). The Trexo requires a person outside of the user to facilitate steering and turning via the guide bar attached to the Rifton frame. For this study protocol, the Trexo robotic legs will be oriented outward (facing out of the open side of the Rifton Pacer frame), to maximize the child’s hands-free participation during physiotherapy (Tao et al., 2020).

#### 2.4.2 Physiotherapy team training and treatment strategy

The Trexo device will be a new physiotherapy intervention tool for physiotherapists. As such, to ensure safe, effective, and competent use that aligns with the child’s abilities and individualized gait goals, PT/PTA training will include: (i) vendor-created technical materials and virtual training from the Trexo company; (ii) in-person shadowing of Trexo use at a community clinic; (iii) motor-learning strategies online educational materials created at our center and based on the Motor Learning Strategies Rating Instrument (Ryan et al., 2019, 2020; Spivak et al., 2021); (iv) Trexo piloting with typically developing children; and (v) ongoing peer-mentoring process within our center.

**FIGURE 3:**
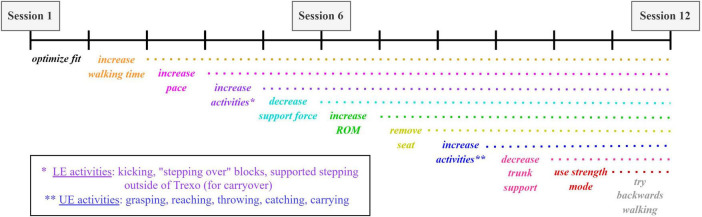
Sample of physiotherapy progression throughout duration of 12 Trexo intervention sessions. Choice of incremental challenges (through Rifton or exoskeleton adjustments) can be tailored to the participant and their individual response to therapy.

Trexo physiotherapy sessions will use an incremental progression process consistent with a motor learning approach, with increasingly challenging tasks presented over time (Figure 3). Participants may also perform exercises (i.e., stretching) to a maximum of 10 additional minutes at the start of the session that may be helpful in these sessions to facilitate the child’s comfort in the Trexo. Principles of motor learning (intensity, repetition, variability, task-specificity, etc.), and the parent’s and PT’s structured goals (described in section “2.6.2 Functional priority goals”) will be prioritized during sessions. In each session, the PT/PTA team will work together with the child and Trexo. The PT/PTA will self-select between the roles of (i) steering the exoskeleton and (ii) operating the Trexo tablet and motivating the child. A safety monitoring plan will be in place throughout each Trexo session to mitigate and/or respond to any adverse events (section “2.10 Adverse events”).

#### 2.4.3 Outpatient physiotherapy program

Participants in the outpatient cohort will receive two individual Trexo-based physiotherapy sessions per week (30-min sessions active treatment time excluding initial Trexo set-up time). The session will be led by one of the study’s Trexo-trained PTs along with a Trexo-trained assisting PTA. These clinicians will not necessarily be previously familiar with the child, being assigned instead according to family and PT/PTA availability. To ensure safe and effective communication with the child, parents will be present at all sessions and will help interpret communication and fatigue as needed while the child is in the Trexo. For each session, 60 min will be allocated to setup, Trexo-based physiotherapy, and session feedback documentation by the PT/PTA. Sessions will occur in the center’s gait lab, hallways, or outdoors in the summer months.

#### 2.4.4 In-school physiotherapy program

Participants in the school cohort will receive two Trexo-based sessions per week (20-min sessions active treatment time excluding initial Trexo set-up time) from their school-affiliated physiotherapy team, to be integrated within a typical school week. The PT/PTA feedback documentation will be done at the end of the school day. One Trexo session will be done in gym class (participation opportunities for peer-based activities), and one done individually in the school’s activity center. Parents are not typically present at regular physiotherapy sessions in this school setting. Thus, the child’s usual school PT will provide the Trexo intervention to ensure that the child has a familiar service provider. This PT will have undergone the Trexo user training. The Trexo-trained PTA for these sessions will be assigned based on availability and will not necessarily be previously known to the child.

### 2.5 Feasibility and acceptability indicators

Feasibility and safety will be quantitatively measured by a set of key process, management, and resource indicators (Tickle-Degnen, 2013; Ameis et al., 2020; Ayoub et al., 2020; Hilderley et al., 2022) with associated targets set for this study protocol. A priori targets for feasibility and acceptability are: (i) ability to enroll 80% of eligible participants that are invited to participate in the study; (ii) study retention rate of ≥ 90%; (iii) completion of awake MRIs ≥ 50%; (iv) tolerance (of setup and wear) and retention of muscle recording sensors during walking tests ≥ 75%; (v) tolerance and retention of heart rate sensor chest strap wear during gait (Trexo walker and regular walker) and motor/functional assessment ≥ 75%; (vi) tolerance of motor/functional assessments ≥ 90%; (vii) completion of parent and PT/PTA interviews ≥ 75%; (viii) adverse events “mild” severity at most and occur in ≤ 10% of physiotherapy sessions; (ix) Trexo set-up time and pauses due to adjustments significantly decrease over 12 physiotherapy session duration; (x) Trexo software or device glitches occur in ≤ 5% of sessions; (xi) motor learning strategies used “often” (25–49% of time) by PT/PTA team during physiotherapy sessions; (xii) child comfort and task enjoyment self-ratings during physiotherapy sessions reflect ≥ 80% positive scoring; (xiii) perceived study benefit by parent/clinician ≥ 80% positive scoring; and (xiv) PT/PTA training and session satisfaction ratings ≥ 80%.

#### 2.5.1 Recruitment, retention and adherence

Recruitment rate will be calculated as the percentage of participants enrolled as a function of the number of participants invited. Study retention will be calculated as the enrolled participants who complete all required parts of the study as a function of total enrolled participants. Tolerance will be defined as procedural adherence for each methodology, whereby completion of each of the various measurement and intervention components will be carefully considered for planning of future interventions. The acceptability of physiotherapy intervention frequency of twice per week will be assessed based on attendance and family feedback.

#### 2.5.2 Safety

Potential risks for Trexo physiotherapy may include the usual risk of muscle soreness when doing walking-based activities, skin irritation from the exoskeleton’s shin cuffs or other parts of the Trexo’s walking frame, or falling when transitioning child in/out of the Trexo device. Of note, there were no adverse events documented during a 3-month case study of Trexo use at home and in the community (Diot et al., 2021).

To support close monitoring of any negative physical impacts associated with Trexo use, the treating PT will document the presence of any skin irritation experienced by the participant before and immediately after each treatment session. If pain is indicated by the child, the PT will check the identified area for bruising, redness or other skin/tenderness issues that might be associated with exoskeleton wear and/or the previous Trexo session. The child’s own method of discomfort/pain communication will be taken into account when quantifying the discomfort. Body location, discomfort type (muscle vs. skin), severity (mild to severe), duration (temporary vs. sustained) of pain will be tracked thoroughly, with recommendations made for next steps (section “2.10 Adverse events”). These safety categories will be summarized when assessing the acceptability of the Trexo intervention.

#### 2.5.3 Session tracking and documentation

All of the Trexo-based physiotherapy sessions will have associated content summary sheets (completed by the treating PT) that will permit systematic documentation of motor tasks undertaken, successes, challenges, adjustments, strategies applied, and next session planning. Additionally, a non-PT member of the research team will document the following at each session: attending staff roles, extra equipment/props used, Trexo setup time, Trexo tablet modes used, changes to range of motion or robotic support force, physical adjustments, walking and standing activities, total step count and walking time, timing and rationale for all session pauses (i.e. rest, adjustment, discomfort, standing activity), socialization opportunities, child communication modes, and observable fatigue (Supplementary Material 1).

Documentation of the active ingredients of PT interventions using exoskeleton treatments (i.e., device parameter changes and activities undertaken) has been largely missing in the literature to date. There is strong advocacy now to include tracking and reporting of device usage parameters in future trials to aid in the understanding of how best to apply the technology and better support the translation of best practice protocols into clinical practice (Cherni and Ziane, 2022; van Dellen and Labruyère, 2022). Thus, one session per child (the 10*^th^* or 11*^th^*) will be videorecorded to allow documentation of the extent to which motor learning approaches were taken by the PT/PTA team, assessed by an external rater using the *Motor Learning Strategies Rating Instrument* (MLSRI; Spivak et al., 2021).

#### 2.5.4 Child, parent, and physiotherapy team satisfaction

At the end of each session, child-rated Trexo session satisfaction will be assessed using a picture-format (smiley-o-meter based) rating scale (Zaman et al., 2013). The child will be given picture scales formatted as a choice of a happy/neutral/sad face to rate how they felt in the Trexo (comfort), level of exertion (tiredness at end of session), and their level of enjoyment for each of the Trexo activities done. Summaries of each experience category (comfort, tiredness, task enjoyment) will be quantified across all 12 sessions for each participant.

Optional qualitative interviews will capture parent and PT/PTA team perspectives on the Trexo use with the child (section “2.7.2 Postural control assessments”). This will be an opportunity to solicit feedback on study design, tolerance, and opinions on different study aspects from a clinical and family perspective. PT/PTA teams will have the opportunity to reflect on the Trexo training process and the rollout of the intervention itself, so that this process can be optimized for future PT/PTA cohorts.

### 2.6 Primary study outcomes

Primary study outcomes will be assessed at multiple timepoints (Table 2) pre- and post-intervention.

**TABLE 2:** Outcome measures across timepoints.

		Timepoint			
Measures	Completed by	O_0_	X	O_1_	O_2_
**Primary outcome measure**					
GMFM-88*	PT assessor	•		•	•
COPM *	Parent with PT assessor	•		•	•
GAS	Treating PT/PTA		**T** (†); **T** (‡)		
MRI	SR with MRI technician	•		•	•
MMG	SR		**T, W** (†); **T, W** (‡)		
**Secondary outcome measure**					
ROM and Tardieu Spasticity*	PT assessor	•		•	•
PPAS and LSS*	PT assessor	•		•	•
PEDI-CAT*	Parent	•		•	•
1WT and DMA	Treating PT/PTA		**T, W** (†); **T, W** (‡)		
Heart rate	SR		**T, W** (‡)		
**Participant characterization**					
MACS*	PT assessor with parent	•			
CFCS	Treating PT and SR			**T**	
Dimensions of mastery*	Parent	•			
Trexo physical adjustments	Treating PT and SR		**T**		
**PT and parent perspectives**					
Parent baseline interview	Parent with SI		•		
Parent outcomes interview	Parent with SI			•	
PT training interview	Treating PT/PTA with SI			•	
PT outcomes interview	Treating PT/PTA with SI			•	

*Completed during motor/functional assessment visit. **T** = done in Trexo; **W** = done in regular walker; • = done without any device. (†) denotes the beginning of the intervention phase and (‡) denotes the end of the intervention phase.

#### 2.6.1 Gross motor skills

The *Gross Motor Function Measure* (GMFM-88; Russell et al., 2000) will be administered by an independent PT assessor during the motor/functional assessment at baseline and immediately post-intervention. It will also be used 1-month post-intervention to gauge maintenance/progression of any functional gains. PT assessors will be trained on the GMFM-88 and all other PT assessment measures by the study’s co-principal investigator prior to their first study assessment. All PTs will already have extensive clinical experience administering the GMFM with children with CP, and variable experience with the other measures. The full 88-item version of the GMFM will be used as it more comprehensively captures a range of abilities in its lowest dimensions (Lie/Roll and Sit) than the abbreviated GMFM-66. This will be important as better trunk and head control are anticipated to be goals and potential outcomes associated with Trexo use in children in GMFCS Level IV. The GMFM-88 testing will be captured on video to enable post-assessment review by PTs. Pre/post change scores will be calculated with descriptive statistics representing results of the group. The GMFM has excellent test-retest reliability (Bjornson et al., 1998), and responsiveness to change (Vos-Vromans et al., 2005).

#### 2.6.2 Functional priority goals

The PT assessor will guide the attending parent(s) through the *Canadian Occupational Performance Measure* (COPM; Law et al., 1990) during the baseline functional assessment visit to set priority outcome goals for their child. These 3–4 goals will be guided from a menu prepared by the investigators of walking-based or other functional outcomes that may arise from Trexo use. These goals can relate to the child’s abilities in home, school, or community environments. The parent(s) will use the COPM’s 10-point response scales to rate the importance of each goal, as well as satisfaction and performance of each at baseline. COPM goals will be rated by the parent(s) again at the post-intervention assessment, as well as the 1-month post intervention assessment. All efforts will be made to have the same parent attend each assessment to maintain consistency. The parent(s) and assessor will be blinded to previous COPM scores. Pre/post mean change scores will be calculated for each child. The COPM has been adapted for use with children and has demonstrated strong internal consistency, construct validity, and responsiveness to change (Cusick et al., 2007).

*Goal Attainment Scaling* (GAS) goals and achievement levels will be set by the treating PTs, and will be Trexo-directed or regular walker-associated. GAS will serve as an individualized measure of change for each child and will link to the parent-chosen COPM goals to allow evaluation of the targeted subcomponents of COPM’s higher level functional priority goals (King et al., 2000; Ostensjo et al., 2008). These two or three goals will be set by the 3*^rd^* physiotherapy session, allowing the PT team to first form a realistic idea of the child’s functional abilities and potential areas of improvement. GAS outcomes will be scored per goal by the child’s treating PT upon completion of the child’s last physiotherapy session, and a summary T-score will also be calculated. GAS is commonly used with children with CP who use walkers, and has demonstrated strong internal consistency, construct validity, and responsiveness to change (Paleg and Livingstone, 2016).

#### 2.6.3 Neuroimaging

**FIGURE 4:**
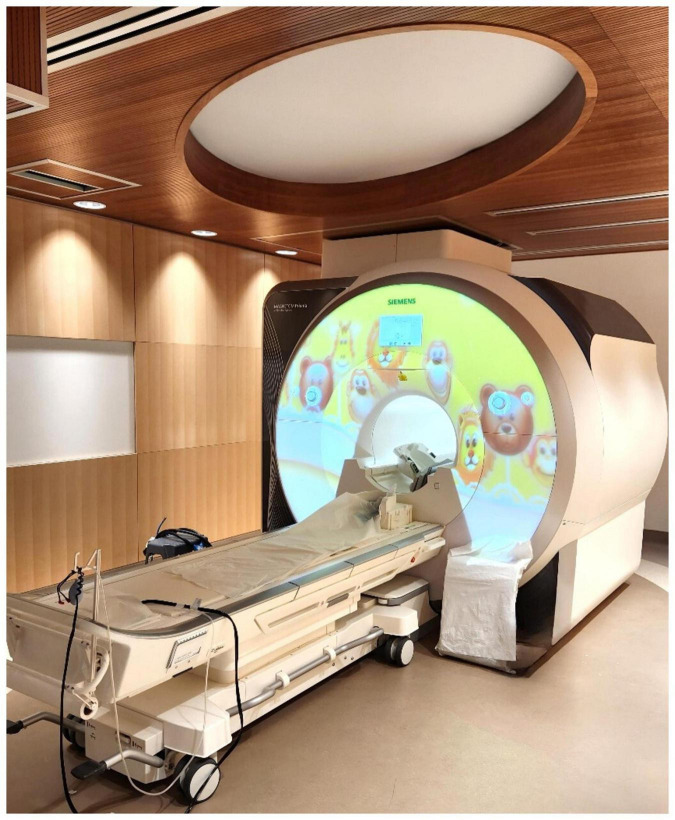
The MRI suite at Holland Bloorview Kids Rehabilitation Hospital (Toronto, Canada). This suite is fully accessible, child-friendly, research-focused, immersive, and customizable. Awake MRI head scanning occurs pre- and post-intervention.

Awake magnetic resonance imaging (MRI) head scanning will be done in the Siemens Prisma MAGNETOM 3-Tesla MRI scanner with a 36-channel head coil at Holland Bloorview Kids Rehabilitation Hospital (Figure 4). If tolerated, these will be done at baseline and immediately post-intervention, with an option for a third scan 1-month post-intervention. Prior to the baseline scan, families will be provided with MRI resources intended to prepare and educate them for an awake scan with their child. Resources include a child-friendly MRI explanation book, video links to a tour of the center’s MRI suite, links to MRI cartoons, links to MRI sound samples, and a link to the resting state fMRI video. This is meant to ease any apprehension or stress associated with the MRI process.

Total MRI acquisition time will be kept under 40 min for this young demographic. MRI scanning will include T1-weighted scanning, T2-weighted scanning for incidental findings, diffusion kurtosis imaging (DKI), and resting state functional MRI (fMRI) (scan parameters: Supplementary Material 2). Participants will wear noise-canceling headphones and watch videos of their choice during the structural scans, and the visual paradigm Inscapes video (headspacestudios.org/inscapes) during the fMRI scan. Incidental reviews of all scans will be completed by a pediatric radiologist.

#### 2.6.4 Muscle recordings

Mechanomyography (MMG) muscle recordings (Plewa et al., 2017) from each child will be recorded at baseline and post-intervention, concurrently with walking assessments (section “2.8.3 Motivation-related traits”) in their regular maximum support walker (if they have one) and the Trexo walker.

**FIGURE 5:**
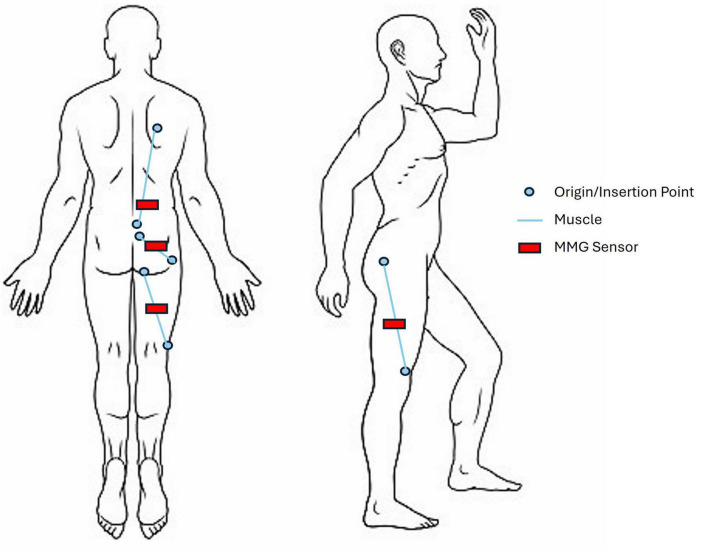
Mechanomyography sensor layout, shown on the right side of the body. Target muscles include vastus lateralis, biceps femoris (long head), gluteus maximus, and erector spinae (longissimus thoracis). Sensors will be secured on top of the skin with medical tape and located according to origin and insertion points of the muscles.

Bilateral MMG data will be collected using tri-axial ADXL335 accelerometers (2.0 cm x 1.5cm; sampling rate 1000 hz/channel), powered by a 3.3V regulator. 8 accelerometers will be placed on muscle sites bilaterally to record muscle vibrations: erector spinae (longissimus thoracis), biceps femoris, vastus lateralis and gluteus maximus. These muscles were selected based on their role in gait (Hesse and Uhlenbrock, 2000; Jonkers et al., 2003; Sousa and Tavares, 2012; Di Nardo et al., 2015) and body surface accessibility for muscle sensors while in the Trexo. Accelerometers will be attached with medical tape on the skin above the largest part of the muscle belly (Figure 5). All selected muscle sites are above the knee as Trexo shin cuffs preclude access to below-knee locations. An additional (9^th^) sensor will be attached to the knee joint of the Trexo device to record robotic leg movement.

Each accelerometer will be wired to a data collection unit comprised of two National Instruments USB-6210 data acquisition cards. The data acquisition cards will plug into a laptop computer via USB, which will be fitted into a backpack that travels with the walkers (hung on the back of the Trexo or carried by a member of the research team). A second computer will connect remotely to the laptop, allowing the researcher to start and stop the data collection as well as remotely mark the data in real time during significant events (e.g., pauses for adjustments, start/stop of walking assessments).

MMG data collection will take place in 25-min testing sessions (including setup; per walker) that will encompass the 1-min walk and dynamic mobility assessment (section “2.7.4 Gait assessments”) followed by a free (unstructured) walking period with the child. Each participant will walk for a minimum of 5 min (or until fatigue to a maximum of 20 min) in their regular maximum support walker and the Trexo exoskeleton. Baseline MMG readings will be taken during quiet standing for 30 s at the start of each testing session – these readings will be used to normalize signals.

### 2.7 Secondary study outcomes

Secondary study outcomes will be assessed at multiple timepoints (Table 2) pre- and post-intervention.

#### 2.7.1 Body structure and function

*Passive range of motion* (ROM) of selected movements (hip flexion contracture; hip abduction; popliteal angle; knee flexion contracture; ankle dorsiflexion with knee flexed or extended), and evaluation of resting and dynamic spasticity as measured by the *Tardieu Scale* (Scholtes et al., 2006) at knee and ankle (hip adductors, hamstrings, gastrocnemius with knee flexed and extended) will be conducted at all functional assessment timepoints. This is important to establish at baseline (excerpted from screening session data where ROM eligibility was confirmed) to monitor adverse events, and permit evaluation of changes in ROM associated with Trexo use.

#### 2.7.2 Postural control assessments

Two postural assessments will be administered by the physiotherapist assessor, and scored from video that will be captured during the assessment by a research assistant using a standardized camera angle protocol.

The *Posture and Postural Ability Scale* (PPAS; Rodby-Bousquet et al., 2016) is a validated measure for children with CP in GMFCS levels II to V. It will rate the symmetry and alignment of the child’s head, trunk, pelvis, legs, arms, and weight distribution in frontal and sagittal planes. The child will be guided into prone, supine, sitting and supported standing positions (30 s in each) by the assessor. Scoring will be done from the video of the assessment.

The *Level of Sitting Scale* (LSS) (Fife et al., 1991; Field and Roxborough, 2011) is designed for children who are wheelchair users and require some degree of external support (GMFCS IV and V). The LSS will classify a child’s sitting ability without feet supported. It will serve as both a descriptive measure of children enrolled in the study and also an outcome measure in tandem with the PPAS. It will be scored from the PPAS video.

#### 2.7.3 Functional abilities parent-report questionnaire

The *Pediatric Evaluation of Disability Inventory* (PEDI-CAT) (Haley et al., 2011; Shore et al., 2017) parent-report questionnaire’s Daily Activities (speedy version), Mobility (content version) and Social/Cognitive (speedy version) domains will be completed by the child’s parent at each of the functional assessments, via a secure weblink on a study tablet. If both the parents are present, they may complete it together.

#### 2.7.4 Gait assessments

Functional gait will be assessed via a *1-minute walk test* (1MWT) (Hassani et al., 2014) and the *Directional Mobility Assessment* (DMA) (Wright and Jutai, 2006; Livingstone and Paleg, 2016), done in the child’s regular walking device (if they use one) and the Trexo walker. The 1MWT will measure the distance walked down a straight wide hallway within 60 s with the Trexo and with the child’s regular walker, with support from the child’s PT/PTA team as needed for steering (Trexo and regular walker) and facilitating steps (regular walker). The DMA incorporates a functional walking course: straight walking, turns, obstacles, narrow path, and backing up.

These walking tasks will be administered by the child’s treating PT/PTA simultaneously during MMG/heart rate monitoring (facilitated by study researchers). Involvement of the PT/PTA in this testing is essential as they are Trexo-trained and aware of the child’s functional abilities and safety considerations. A video recording will be made of these walking tests to permit their rating by an independent PT assessor to maintain independence of scoring from the clinical team.

#### 2.7.5 Heart rate

*Heart rate* during gait will be captured using a Polar H10 Heart Rate Sensor (wireless chest strap placed around the torso) which will transmit data (beats per minute, RR intervals, and heart rate variability taken continuously) via Bluetooth to a secure device. Heart rate will be captured post-intervention (end of Phase X) concurrently with muscle recordings (section “2.6.4 Muscle recordings”) during Trexo and regular walker use at the timed walk and DMA assessments since robotically facilitated walking may impact heart rate differently than would a manual walker. Resting heart rate will be captured initially in each instance. Heart rate will also be captured in the absence of walking, during the post-intervention functional assessment.

### 2.8 Participant characterization

The following assessments will be completed only once during the child’s study participation as they portray a fixed trait/ability (i.e., not outcome measures). These assessments will help characterize each participant and may serve as predictors or correlates for feasibility and outcome measure results.

#### 2.8.1 Upper extremity function

The *Manual Ability Classification System* (MACS or MiniMACS; Eliasson et al., 2006) will be completed by a PT assessor at the end of the baseline motor/functional assessment as they reflect the child’s hand function observed during upper and lower body tasks in the assessment and confirm child’s day to day hand function in conversation with the parent- at the assessment. Children in GMFCS IV may have hand function at a different level than that of their gross motor function, and these hand and arm abilities may have an impact on what the child will be able to do during Trexo physiotherapy. Hence this information may be important in the interpretation of sessional data and ultimate goal accomplishment.

#### 2.8.2 Communication ability

The *Communication Function Classification System* (CFCS; Hidecker et al., 2011) will be used after all 12 Trexo physiotherapy sessions have been completed, to capture the child’s communication style over time while in the Trexo.

#### 2.8.3 Motivation-related traits

The *Dimensions of Mastery* questionnaire (Morgan et al., 2006; Igoe et al., 2011) will be completed by parents at the baseline motor/functional assessment only. This information about the child’s underlying motivation traits may help identify personal characteristics related to engagement and learning that maybe contribute to Trexo session success.

#### 2.8.4 Individualized Trexo physical adjustment templates

Each child will have their own baseline Trexo adjustment template (Supplementary Material 3) created during their pre-physiotherapy fitting session and updated through the course of the child’s Trexo use. This template will compile Rifton Pacer settings (chest prompt size/tilt, Rifton frame height, seat height, seat angle, and seat position), Trexo leg settings (robotic leg width, knee-to-floor length, hip-to-knee length, and footplate size), and Trexo tablet settings (range of motion and optimal support forces for hip and knee joints). These settings will be selected by the treating PT to ensure good postural alignment, comfort, and adequate heel strike during walking. Since a single Trexo device will be shared across the children in the study, the research team will refer to this profile prior to each child’s physiotherapy session to make sure all child-specific physical adjustments are made to the Trexo before the session starts.

### 2.9 PT and parent perspectives

Qualitative interviews will be scheduled separately with the PT/PTA team and parents with an independent study interviewer using semi-structured interview guides. These interviews will be scheduled proximal to the pre- and post-intervention study timepoints.

Participating PTs and PTAs will have the opportunity to share their perspectives via two optional interviews: (i) reflection on the strengths and limitations of the Trexo training process used following the completion of their training and a round of Trexo physiotherapy sessions for at least one child (∼30–60 min); and (ii) Trexo user experience for each participant, following the end of that child’s physiotherapy intervention (60–90 min).

Parents’ study expectations and goals will be collected at two optional interview timepoints: (i) baseline; about Trexo outcomes goals and hopes (∼20 min); and (ii) post-Trexo intervention; focusing on observed outcomes, impressions about study methodologies, and individualized goal accomplishment including questions about overall quality of life in addition to the questions about mobility (∼45 min). Since the research team will see the child for only 2 h a week, parent feedback will be essential to capture functional changes observed in the daily living context of their child. This will also be an opportunity to solicit parental feedback on study design, tolerance, and opinions on different study aspects.

### 2.10 Adverse events

In the case of an adverse event, the study physician will be contacted for the recommended course of action, which will depend on the severity and circumstances of the adverse event. Documentation of the event will be completed by the treating PT, principal investigator, and study physician as per the study’s adverse event form. A treatment plan will be made at the discretion of the study physician. If the study team concludes that the Trexo presents a possible continuing risk to the child, Trexo treatment will be discontinued for that child and the post-Trexo outcomes assessment will be completed at that time. Additionally, the parent or child can decide to discontinue the study at any point for any reason. They will be invited to complete the next follow-up assessment at that point but are free to decline this as well. If any children drop out of the study, no children will be recruited in their place since this discontinuation of participation could point to an underlying acceptability or safety issue.

### 2.11 Data analysis

#### 2.11.1 Quantitative data analysis

*Feasibility/safety/acceptability indicators* (section “2.5 Feasibility and acceptability indicators”) will be summarized for each child’s sessions and the group descriptive statistics will be compared to *a priori* target values.

*Motor/functional assessment measures and functional priority goals* will have total/dimension scores summarized via descriptive statistics. Paired *t*-tests or non-parametric equivalents will be conducted for baseline and post-intervention time points for the co-primary measures (GMFM-88 and COPM goals), and then with each of the secondary outcomes. Correlational analyses (Pearson’s r) will also be undertaken to investigate associations between primary and secondary outcomes or with MRI results. Each clinical measure will be graphically inspected for any patterns from baseline to the optional 1-month post-intervention timepoint. For a subsample of children (undetermined subgroup ‘n’ at this point), comparisons will also be made between walking performance in the child’s regular walker and the Trexo at baseline and post-intervention.

*MRI scans* will be processed with Freesurfer Software (surfer.nmr.mgh.harvard.edu), employing a longitudinal pipeline. Automated segmentation of structural T1-weighted images will be performed, followed by quality control steps to ensure image clarity. Any neuroimaging data with excessive motion artifact will be excluded from the study. Regions of interest (ROI) will be selected based on brain areas that are most associated with gait, motor function, and motor learning. From T1 anatomical data, mean cortical thickness per brain region, and white and gray matter volumes will be calculated. Diffusion data will be used to identify and reconstruct relevant white matter tracts as well as derive DKI and DTI mean metric maps, kurtosis fractional anisotropy (FA) values, and mean kurtosis (MK), as outcome measures. From the fMRI data, ROI-specific time courses of the BOLD signal will be computed by averaging time courses across voxels within each ROI. fMRI outcome measures will include functional connectivity (FC) correlation matrices and a structural-decoupling index.

*MMG* signals will be calculated as the magnitude of the vector sum of the tri-axial ADXL335 accelerations. Data will be segmented according to periods of activity and normalized to the initial period of quiet standing for each participant. Accelerometer data will be processed using Matlab Software (Natick, Massachusetts: The MathWorks Inc.): bandpass filtered between 5 and 100 Hz (4^th^ order Butterworth filter) and processed with a symlet wavelet transform (Alves and Chau, 2010; Achmamad and Jbari, 2020). Device-induced noise will be compared in the Trexo and the child’s regular walker. Heel strike will be identified based on video recordings to demarcate phases of the gait cycle and changes in magnitude and slope of the force. We will extract and select features in time, frequency, and time-frequency domain for classification.

#### 2.11.2 Qualitative data analysis

Anonymized individual physiotherapy summary profiles will be created from the session documentation, detailing goals worked on, Trexo settings used, activities undertaken, things that went well, and challenges that presented during sessions. Collective data profiles across children from their first, mid-point, and last sessions will be analyzed via a content analysis approach (settings/activities/challenges) to summarize the operational details related to Trexo use and elucidate any patterns of progression of Trexo settings, walk distances and activities undertaken within the Trexo treatment block.

*All interviews* will be transcribed within Zoom, checked afterward by the interviewer for accuracy and then de-identified prior to thematic analysis. Data will be analyzed using NVivo Software (QSR International Inc., Burlington, Canada), and an inductive content analysis approach will be taken to generate the initial codebook (Braun and Clarke, 2006). Parent and PT/PTA interviews will undergo separate thematic analysis to support within-group development of preliminary codes. Specific wording used by participants will be included in the codes to assist in preserving the meaning participants attribute to their actions and processes (Maher et al., 2018). Research team meetings will advance code-to-category-theme development and propose alternate/refined themes and interpretations until group consensus is reached.

A concurrent mixed methods approach will be taken where qualitative and quantitative data will be presented together by theme, and results reported in a narrative joint display (Fetters et al., 2013; Guetterman et al., 2015). Transferability of the results will be facilitated by reporting relevant study participant demographics to contextualize the findings.

## 3 Discussion

Overground exoskeleton use offers children who have restricted ambulatory abilities the opportunity to uniquely access their surrounding environment within a device that provides safety and stability (Diot et al., 2021) while also offering the opportunity to incorporate hand and arm use during walking-based activities. To our knowledge, this will be the first study to investigate feasibility indicators, outcomes, and experiences of Trexo-based physiotherapy in school and outpatient contexts for children with severe mobility impairments. Our rehabilitation protocol is based on motor learning principles that may promote experience-dependent neuroplasticity (Gassert and Dietz, 2018; Berger et al., 2019) and changes in functional, neural, and muscular outcomes.

Compared to conventional gait training, the preparation and execution of physiotherapy treatment blocks using new gait technologies is more cognitively demanding for PTs (Read et al., 2020; Ouendi et al., 2022; van Dellen et al., 2023; Murphy et al., 2024). This added aspect underscores the importance of evaluating the feasibility and acceptability of the “typical” Trexo session itself. In our pediatric context, quantitative and qualitative data captured during Trexo physiotherapy sessions will provide user-based information that may address questions related to clinical adoption and utility from the PT perspective. Specifically, stakeholder feedback (child, parents, clinician) from interviews and feasibility/session data overall will guide the creation of future training materials, and evidence-based implementation protocols for PTs. It will also facilitate realistic goal setting and capture impressions of parents about the value of this technology for their preambulatory children and any extended associated impact on performance in routine activity or on quality of life more broadly.

Understanding the body-wide outcomes associated with gait training in children with CP is essential for safety and maximizing positive functional results. The characterization of therapy-dependent neuroplasticity (Peters et al., 2020) may provide indications about how responses to exoskeleton-assisted gait therapy may have some association with a child’s neurological profile (Schwartz and Meiner, 2015), and may be associated with different changes in functional and muscular behavior (Snodgrass et al., 2014; Sczesny-Kaiser et al., 2015; Perpetuini et al., 2022). Quantifying muscle behavior and heart rate during robotic gait training can help establish any training limit thresholds that might need to be put in place to prevent injury and over-exertion in physiotherapy treatments (Brunton and Rice, 2012; Puce et al., 2021), especially for nonverbal children. Future overground exoskeleton development can be paired with this knowledge of body-wide outcomes to advance mechanical feedback responses to the child’s physiological signals, thereby integrating with the child’s existing motor abilities while also compensating for skill deficiencies.

This study will contribute evidence-based knowledge to guide clinical decisions about the introduction of the Trexo or similar lower-limb exoskeletons within pediatric rehabilitation settings, and serve as an empirical foundation for a progressive program of multi-center research. In addition to the field of CP, this research could be broadened to include individuals with other non-progressive neuromotor conditions which impair the lower body, including those in GMFCS V (non-ambulatory children), if adequate safety and acceptability are established.

## 4 Ethics and dissemination

### 4.1 Ethics approval and consent to participate

This study protocol was approved by the research ethics board of Holland Bloorview Kids Rehabilitation Hospital (no. 0523), and the University of Toronto (no. 00044118), according to Resolution 466/12 of the National Health Council and the Declaration of Helsinki. Researchers will invite children and parents to participate voluntarily and sign the informed consent forms to be included in the study.

### 4.2 Consent for publication and confidentiality

Participation information will be kept confidential and stored securely in the hospital database. Only researchers will access the database, ensuring anonymity, respect, and human dignity. Results will be published in peer-reviewed journals and presented at scientific meetings. In case of significant changes in the protocol, we will inform participants, Clinicaltrials.gov, and journals. If requested, we will provide a copy of the informed consent form.

### 4.3 Availability of protocol and data

This protocol information is registered and available: [https://clinicaltrials.gov], identifier NCT05463211. The corresponding author will provide the study protocol and data on reasonable request to achieve study goals.

## Ethics statement

The studies involving humans were approved by the Research ethics committees at Holland Bloorview Kids Rehabilitation Hospital REB (no. 0523), University of Toronto (no. 00044118). The studies were conducted in accordance with the local legislation and institutional requirements. Written informed consent for participation in this study was provided by the participants’ legal guardians/next of kin.

## Author contributions

SB: Conceptualization, Data curation, Formal analysis, Funding acquisition, Methodology, Project administration, Resources, Validation, Visualization, Writing – original draft, Writing – review and editing. LH: Writing – original draft, Writing – review and editing. TC: Conceptualization, Funding acquisition, Methodology, Resources, Supervision, Writing – original draft, Writing – review and editing, Validation. FVW: Conceptualization, Funding acquisition, Methodology, Resources, Supervision, Writing – original draft, Writing – review and editing.

## Footnotes

1
www.rifton.com
