# Supplementary material for: Physiotherapy-assisted overground exoskeleton use: mixed methods feasibility study protocol quantifying the user experience, as well as functional, neural, and muscular outcomes in children with mobility impairments
Source: Front Neurosci. 2024 Jul 31;18:1398459. doi: 10.3389/fnins.2024.1398459 (PMC11322617; doi:10.3389/fnins.2024.1398459)
Supplement: Supplementary file 1 [file Data_Sheet_1.PDF]

Supplementary Material

Supplementary Material 1. Trexo Session Forms: "Trexo Session Tracking" forms will be completed by study researchers attending the Trexo physiotherapy intervention sessions. The "PT/PTA Summary Log Sheet" and "Session Activity Tracking Log" will be completed by treating PTs/PTAs. Each form is completed per session.

Supplementary Material 2. MRI Scan Parameters: structural, diffusion, and resting state functional scans.

	**3D T1**	**3D T2**	**dMRI**	**fMRI**
**Sequence**	3D MPRAGE	SPACE	Echo planar (b=1000,1600, 2600 mm^2)	EPI BOLD
**Field of view (mm^3)**	256	256	244	222
**Slice orientation**	Sagittal	Sagittal	Transversal	Transversal
**TE (ms)**	1870	409	73	30
**TR (ms)**	3.1	3200	3800	1500
**Flip Angle (°)**	9	-	-	70
**Voxel Size (mm isotropic)**	0.8	0.8	2	3
**Slice Thickness (mm)**	0.8	0.8	2	3
**Number of slices (contiguous)**	240	190	70	50
**Directions**	-	-	36, 46, 67	-
**Scan time**	5 min 03 s	7 min 22 s	2 min 35 s, 3 min 13 s, 4 min 33 s	5 min 10 s

Supplementary Material 3. Trexo Physical Adjustment Template: to be completed during the Trexo fitting/acclimation session(s), pre-intervention. This will include measurements and first steps in the Trexo to ensure comfort of fit.

Trexo Session Tracking

Staff present/role in the session: 1.____ 2.____
3.____ 4.____

Session start-time: _______ Start time once set-up completed: ________ End of walking in Trexo time: _________
Individual session: _____ OR Gym/group session: ______ Location of session: _________________

1.Start time: 0:00 *Pace (steps/min): ______ Pace or other setting changes while moving: ______________________________________
*Stop time: _______ *Total Steps Taken: ______ Reason for stop: ________________________________Time paused (from stop watch): ______
Physical activity done: ____________________ Adjustments made while stopped: _____________________________________

2.Start time: 0:00 *Pace (steps/min): ______ Pace or other setting changes while moving: ______________________________________
*Stop time: _______ *Total Steps Taken: ______ Reason for stop: ________________________________Time paused (from stop watch): ______
Physical activity done: ____________________ Adjustments made while stopped: _____________________________________

3.Start time: 0:00 *Pace (steps/min): ______ Pace or other setting changes while moving: ______________________________________
*Stop time: _______ *Total Steps Taken: ______ Reason for stop: ________________________________Time paused (from stop watch): ______
Physical activity done: ____________________ Adjustments made while stopped: _____________________________________

4.Start time: 0:00 *Pace (steps/min): ______ Pace or other setting changes while moving: ______________________________________
*Stop time: _______ *Total Steps Taken: ______ Reason for stop: ________________________________Time paused (from stop watch): ______
Physical activity done: ____________________ Adjustments made while stopped: _____________________________________

5.Start time: 0:00 *Pace (steps/min): ______ Pace or other setting changes while moving: ______________________________________
*Stop time: _______ *Total Steps Taken: ______ Reason for stop: ________________________________Time paused (from stop watch): ______
Physical activity done: ____________________ Adjustments made while stopped: ______________________________________

6.Start time: 0:00 *Pace (steps/min): ______ Pace or other setting changes while moving: ______________________________________
*Stop time: _______ *Total Steps Taken: ______ Reason for stop: ________________________________Time paused (from stop watch): ______
Physical activity done: ____________________ Adjustments made while stopped: ______________________________________

7.Start time: 0:00 *Pace (steps/min): ______ Pace or other setting changes while moving: ______________________________________
*Stop time: _______ *Total Steps Taken: ______ Reason for stop: ________________________________Time paused (from stop watch): ______
Physical activity done: ____________________ Adjustments made while stopped: ________________________________

***Total number of steps taken in this session**: _______ ***Total session walk time on tablet: ____________**

***Cumulative # of steps for all sessions: ________** ***User i**n**itiation** % Left leg: _________ % Right leg: __________

Number of pauses for adjustments/repositioning during session (NOT stops done to allow a standing activity): ______

***Support level: ______________** Any changes in support level during session? No _______ Yes _______

If yes, please indicate new level and when this occurred in the session and comments on rationale for change:

____

Pacing Notes throughout session: ____

Endurance mode or Strength mode: ____

*** Left side ROM settings at start of session:** Hip max ext _____ Hip max flex _____ Knee max flex _____ Knee min flex _____

Any left side ROM changes during session? No _______ Yes _______ If yes, specify new ranges and rationale for change:

____

NEW RANGE: Hip max ext _____ Hip max flex _____ Knee max flex _____ Knee min flex ____ Changed @ Session time: ______

*** Right side ROM settings at start of session**: Hip max ext _____ Hip max flex _____ Knee max flex _____ Knee min flex _____

Any right side ROM changes during session? No _______ Yes _______ If yes, specify new ranges and rationale for change:

____

NEW RANGE: Hip max ext _____ Hip max flex _____ Knee max ext _____ Knee min flex ____ Changed @ Session time: ______

Comments about session pauses, tasks, communication, Trexo training challenges, session highlights, social interactions, fatigue

____

____

____

Recorder's name and Signature: ____ **Date: _____________________**

Child's Study ID: ___________ Treating PT's Initials: ____________ Date: __________________
mm-dd-yyyy

Trexo PT Session Activity Log

- Write each activity in that you did at this session. Then for each activity, place a **'1'** in the box that indicates the **focus of the activity** and a **'2'** in **ALL other boxes** that the activity relates to in <u>your session</u>.
- The **focus of an activity** can change from session to session.
- Activity details should include limb(s) (right/left) and joint(s) as appropriate. Also please note the <u>location</u> where activity took place

**#**	**Activity Details (and location) Individual ____ OR Gym Class _____**	**Duration (min)**	**Trexo walking distance (endurance)**	**Trexo walking speed (fitness)**	**Functional use of Trexo**	**Directional control of the Trexo**	**Quality of Gait in Trexo**	**Neck, Trunk Control in Trexo**
1								
			**Focus on upper limb use in Trexo**	**Focused dual tasking while in Trexo**	**Other (specify in the box)**			
		**Details:**						
		**Duration (min)**	**Trexo walking distance (endurance)**	**Trexo walking speed (fitness)**	**Functional use of Trexo**	**Directional control of the Trexo**	**Quality of Gait in Trexo**	**Neck, Trunk Control in Trexo**
2								
			**Focus on upper limb use in Trexo**	**Focused dual tasking while in Trexo**	**Other (specify in the box)**			
		**Details:**						

Child's Study ID: ___________ Treating PT's Initials: ____________ Date: __________________
mm-dd-yyyy

#	Activity Details (and location)	Duration (min)	Trexo walking distance (endurance)	Trexo walking speed (fitness)	Functional use of Trexo	Directional control of the Trexo	Quality of Gait in Trexo	Neck, Trunk Control in Trexo
3								
			**Focus on upper limb use in Trexo**	**Focused dual tasking while in Trexo**	**Other (specify in the box)**			
		**Details:**						
		**Duration (min)**	**Trexo walking distance (endurance)**	**Trexo walking speed (fitness)**	**Functional use of Trexo**	**Directional control of the Trexo**	**Quality of Gait in Trexo**	**Neck, Trunk Control in Trexo**
4								
			**Focus on upper limb use in Trexo**	**Focused dual tasking while in Trexo**	**Other (specify in the box)**			
		**Details:**						

PT's/PTA's Signature: ________________________________ Date: ____________________

Study Number: ______ **Session Date: ____________**

Trexo: PT/PTA Summary Log Sheet

To be completed after each session

1. **Positive aspects of the session:**

2. **Challenges encountered in the session:**

3. **Specific pain/discomfort/other issues associated with the session:**

4. **Ideas for next session:**

5. **Any follow-up action from today's session:**

6. **Home program ideas/changes**

7. **Other interventions (therapy or medical)/special physical activity since last session:**

PT Signature: _______________________________ **Date:_____________________**

Bloorview RESEARCH INSTITUTE

TREXO PHYSICAL ADJUSTMENT TEMPLATE

Note – settings may change over time.

Participant Profile:
(name on Trexo tablet)

Hip to Knee (cm):

Knee to Floor (cm):

Hip Width (cm):

Trexo Motor Width:

Foot Plate Size:

Shin Cuff Size:

Rifton Height:

Seat Height:

Considerations for Sessions:

Baseline Range of Motion

LEFT LEG

HIP	Max Extension	Max Flexion
KNEE	Max Flexion	Min Flexion

RIGHT LEG

HIP	Max Extension	Max Flexion
KNEE	Max Flexion	Min Flexion

Trexo Support Force:
